# Neoadjuvant Chemoradiotherapy and Larynx-Preserving Surgery for Cervical Esophageal Cancer

**DOI:** 10.3390/jcm9020387

**Published:** 2020-02-01

**Authors:** Kun-Yao Dai, Yu-Chao Yu, Yi-Shing Leu, Chih-Wen Chi, Mei-Lin Chan, Chung-Hsin Tsai, Huan-Chau Lin, Wen-Chien Huang, Yu-Jen Chen

**Affiliations:** 1 Department of Radiation Oncology, MacKay Memorial Hospital, Taipei 10449, Taiwan; kunyaodai@gmail.com; 2 Division of Thoracic Surgery, Department of Surgery, MacKay Memorial Hospital, Taipei 10449, Taiwan; celiaholic@yahoo.com.tw (Y.-C.Y.); mlchan127@gmail.com (M.-L.C.); 3 Department of Otorhinolaryngology, MacKay Memorial Hospital, Taipei 10449, Taiwan; ent.lys@gmail.com; 4 Department of Medical Research, MacKay Memorial Hospital, New Taipei City 25160, Taiwan; cwchid48906003@gmail.com; 5 Department of Nursing, Mackay Medical College, New Taipei City 25245, Taiwan; 6 Division of General Surgery, Department of Surgery, MacKay Memorial Hospital, Taipei 10449, Taiwan; cst4622000@yahoo.com.tw; 7 Division of Medical Oncology and Hematology, Department of Internal Medicine, MacKay Memorial Hospital, Taipei 10449, Taiwan; hcanduhmmh@gmail.com; 8 Department of Medicine, Mackay Medical College, New Taipei City 25245, Taiwan; 9 Mackay Junior College of Medicine, Nursing, and Management, Taipei 11260, Taiwan; 10 Department of Medical Research, China Medical University Hospital, Taichung 40402, Taiwan

**Keywords:** neoadjuvant concurrent chemoradiotherapy (CCRT), esophageal squamous cell carcinoma (SCC), larynx-sparing surgery

## Abstract

Neoadjuvant concurrent chemoradiotherapy (CCRT) followed by surgery is widely used for treating locally advanced esophageal cancer in the thorax. This study evaluated the feasibility of neoadjuvant CCRT as a larynx preservation strategy for treating cervical esophageal squamous cell carcinoma (SCC) by a multidisciplinary team. Fifteen patients with cervical esophageal SCC who received neoadjuvant CCRT and radical surgery at our institution were reviewed. All patients received CCRT using the intensity-modulated radiation therapy with 48 Gy to gross tumor and 43.2 Gy to regional lymphatic basin in 24 fractions. Side effects, clinical tumor responses, pathological responses, and surgical margin status were analyzed. Pathological T down-staging was noted in seven patients (46.7%); pathological complete response was achieved in three patients (20%). Fourteen patients (93.3%) had larynx preservation; eight patients (53.3%) achieved negative surgical margins. The 2-year overall survival, local relapse-free survival, and regional relapse-free survival were 50.6%, 62.2%, and 47.5%, respectively. Neoadjuvant CCRT and larynx-sparing surgery are feasible and tolerable in patients with cervical esophageal SCC. Prospectively designed studies for large patient groups and long-term follow-up results are needed for validating this multimodality therapy.

## 1. Introduction

Esophageal cancer has a high risk of recurrence and metastasis with poor prognosis and is the eighth commonest cancer worldwide, with an estimated 456,000 new cases in 2012 (3.2% of total cancers), and the sixth most common cause of death from cancer, with an estimated 400,000 deaths (4.9% of total deaths) [1]. The 5-year relative survival rate remains <20%, with an age-adjusted mortality rate of 7.4 per 100,000 for males and 1.5 per 100,000 for females [2]. The incidence in Eastern Asia is highest in men (17.0 per 100,000) [1]. As the incidence of esophageal cancer keeps increasing, the clinical investigations remain important.

Cervical esophageal cancer (CEC), which is located between the cricoid cartilage and thoracic inlet, is relatively rare, accounting for approximately 5% of all esophageal cancers [3]. The most common histopathological type of CEC is squamous cell carcinoma (SCC), greater than 90% in the surveillance, epidemiology, and end results database [4]. Whether surgical or non-surgical approaches are optimal is still controversial [5,6]. CEC treatment differs from that of thoracic esophageal cancer because of the complicated structures around the cervical esophagus. Moreover, the metastases of regional lymph nodes for CEC are prone to occur at the upper neck and mediastinum [4]. Radical resection of CEC via pharyngo-laryngo-esophagectomy is the most common method [7,8,9]; however, it is highly likely to cause morbidity and compromise quality of life. Therefore, definitive chemoradiation therapy (dCRT) to preserve the larynx is recommended as the standard treatment modality by the National Comprehensive Cancer Network and European Society for Medical Oncology guidelines [10].

In clinical practice, dCRT for CEC, usually consists of radiotherapy (RT) at 50.4 Gy at 1.8 Gy per fraction followed by a local boost to a total dose of 60–68 Gy [11,12,13,14]. Concurrent chemotherapy generally comprises cisplatin, 5-fluorouracil, carboplatin, and/or paclitaxel. The toxicity of dCRT is moderate to severe with severe dysphagia in 5–10% and some life-threatening adverse events [15,16]. The 2-year overall survival rates of patients with CEC treated with dCRT range from 24 to 66% [11,17], comparable to surgery alone [12,18] and remains unsatisfactory.

Several randomized trials and meta-analyses have demonstrated benefits in survival and tumor down-staging with trimodality therapy in locally advanced thoracic esophageal cancer. Neoadjuvant CCRT followed by surgery has been widely accepted for locally advanced esophageal cancer other than CEC [19,20,21]. Post-CCRT radical surgery with complete resection is a major prognostic factor, indicating the importance of tumor removal [22,23]. Whether this role of surgery in thoracic esophageal cancer can be translated to cervical primary site remains unclear. In this study, we aimed to evaluate the use of neoadjuvant CCRT followed by radical surgery as a larynx-preservation strategy in the treatment of cervical esophageal SCC.

## 2. Experimental Section

### 2.1. Subjects

Data of patients with tissue-proved cervical esophageal SCC received neoadjuvant CCRT between August 2013 and August 2017 at our institution were retrospectively reviewed. All patients underwent pre-treatment evaluations, including physical examination, complete blood count, biochemistry study, panendoscopy with biopsy, esophagogram, chest computed tomography (CT), ^18^F-fluorodeoxyglucose positron emission tomography (FDG-PET), bronchoscopy, and/or endoscopic ultrasonography (EUS). This study was approved by the ethics committee of the Institutional Review Board of MacKay Memorial Hospital (IRB approval number: 18MMHIS061e) for the collection and analysis of all patient information.

### 2.2. Radiotherapy

Radiotherapy was planned based on CT images. Patients were immobilized using a thermoplastic mask (Blessing Cathay Corporation, New Taipei City, Taiwan) or Alpha Cradle^®^ (Smithers Medical Products, Inc. North Canton, OH, USA) in the supine position. The simulation CT scans (Brilliance Big Bore CT simulator/Philips Medical Systems, Cleveland, OH, USA) were obtained using 3-mm-thick slices. The gross tumor volume (GTV) included the esophageal gross tumor and enlarged regional lymph nodes. The clinical target volume (CTV)-48 was defined as 0.5–1 cm outside the GTV. The CTV-43.2 included subclinical mucosal/submucosal disease and high-risk regional nodal basins. Nodal irradiation usually included the cervical lymph nodal levels IV, VI, and paraesophageal and paratracheal lymph nodal regions of the upper mediastinum. The planning target volume (PTV) enclosed the CTV with anisotropic margins at the physicians’ discretion to account for possible uncertainties in patient set-up error or internal organ motion. Intensity-modulated radiation therapy (IMRT) or helical tomotherapy with simultaneously integrated boost (SIB) technique delivered 48 Gy (2 Gy per fraction) to the PTV-48 and 43.2 Gy (1.8 Gy per fraction) to the PTV-43.2. Normal organ constraints were set to limit the proportion of total lung receiving >20 Gy (V20) to 20% (lung V20 < 20%). The maximal dose to the spinal cord did not exceed 45 Gy. The treatment plans were calculated to achieve at least 95% of the PTV being covered with the prescribed dose. RT was withheld for any patient with ≥grade 3 neutrophil count decreased/platelet count decreased (absolute neutrophil count < 1000 cells/μL or platelets < 50,000 cells/μL).

### 2.3. Chemotherapy

All patients underwent concurrent chemotherapy based on platinum analogues (cisplatin or carboplatin). Most patients received weekly cisplatin (30 mg/m^2^ of body-surface area) during the RT course. Chemotherapy was delayed if ≥grade 2 toxicities developed (absolute neutrophil count < 1500 cells/μL or platelet count < 75,000 cells/μL). Cisplatin was administered at 70% of the initial dose if the neutrophil count was 1500–2000 cells/μL or platelets were 75,000–100,000 cells/μL. For three patients with chronic kidney disease, weekly carboplatin with the target area under the plasma concentration-time curve (AUC) equal to 1.5 was administered. Four patients received weekly cisplatin 20 mg/m^2^ and paclitaxel 50 mg/m^2^. Two patients received weekly cisplatin 30 mg/m^2^ and paclitaxel 50 mg/m^2^.

### 2.4. Acute Toxicity and Clinical Response Assessment

All patients were evaluated weekly during CCRT and acute toxicities were graded according to the Common Terminology Criteria for Adverse Events version 4.0. Tumor response was evaluated using panendoscopy, EUS, chest CT, and FDG-PET at 4–6 weeks after the final CCRT. No esophagus biopsy was performed after CCRT. In addition, chest CT images were imported to a radiation planning system and GTV was contoured per the panendoscopy, chest CT, and FDG-PET findings to calculate post-treatment tumor volume. Post-CCRT GTV was compared with pre-CCRT GTV for the treatment response evaluation.

### 2.5. Surgery and Pathological Response Assessment

Before starting CCRT, all patients received a feeding jejunostomy and a subclavian venous-access catheter. After CCRT and re-staging, the surgical procedures generally consisted of right-sided thoracotomy for en bloc esophagectomy followed by abdominal and left cervical approach for reconstruction of the esophagus using proximal gastrectomy and gastroesophagostomy or colon graft via the posterior mediastinal or extrapleural retrosternal route. The method of surgery depends on the proximal end of tumor detected after neoadjuvant CCRT. To avoid persistent aspiration from injury of upper esophageal sphincter by esophagectomy, the indication for larynx-preserving surgery at our institution is that the proximal margin of tumor should be at least 2 cm away from the inferior margin of pyriform sinus of hypopharynx. If the proximal edge of tumor is less than 2 cm away from the inferior margin of pyriform sinus of hypopharynx, pharyngo-laryngo-esophagectomy is performed. In the larynx-preserving surgery group, all patients are resected at 2 cm from the inferior margin of hypopharynx regardless of tumor shrinkage effect after neoadjuvant CCRT. The regional cervical lymph nodes were dissected at the surgeon’s discretion. Pathological response was assessed by pathologists specialized to gastroenterology. Resection was defined as microscopically radical (R0) as a tumor-free resection margin of more than 1 mm. R1 resection was defined as microscopic residual disease or microscopic positive margin (including tumor ≤ 1 mm from any margin).

### 2.6. Follow-Up

After treatment, patients had thoracic/abdomen CT evaluations every 3 months for the first year and every 6 months thereafter until tumor progression. Unscheduled evaluations would be arranged if patients had new or unexplained symptoms.

### 2.7. Statistical Analysis

Continuous data are reported as median and range; categorical data are reported as frequency (percentage). The Kaplan-Meier method was used to estimate the overall survival, local relapse-free survival, and regional relapse-free survival. Analysis was performed using IBM SPSS for Windows statistical software v. 22 (IBM, Armonk, NY, USA).

## 3. Results

### 3.1. Patient Characteristics

Table 1 summarizes the patient characteristics. The median age at diagnosis was 57 years (range, 40–82 years). According to the seventh edition of the American Joint Committee on Cancer staging system, the location of primary cancer site is defined by the position of the proximal edge of the tumor in the esophagus. These patients were diagnosed as CEC by this definition and clinically staged as T1–4b, N0–N3, and M0, stage IIA–IIIC. Some CEC extended to the hypopharynx and/or to the thoracic esophagus, but most patients (18 of 21) had the epicenter of the tumor located in cervical esophagus.

### 3.2. Acute Toxicity of CCRT

Twenty patients received the complete planned CCRT. One patient died at the 21st fraction because of traumatic subdural hemorrhage that was not considered treatment related. Acute toxicities from neoadjuvant CCRT were tolerable (Table 2). The most severe acute toxicities were grade 3 cough, anemia, leukocytopenia, and neutropenia in one patient each (4.8%). The common acute toxicities included grade 1 dermatitis (*n* = 12, 57.1%), grade 1 anemia (*n* = 12, 57.1%), and grade 1 nausea (*n* = 10, 47.6%).

### 3.3. Clinical and Pathological Treatment Response

**Figure 1:**
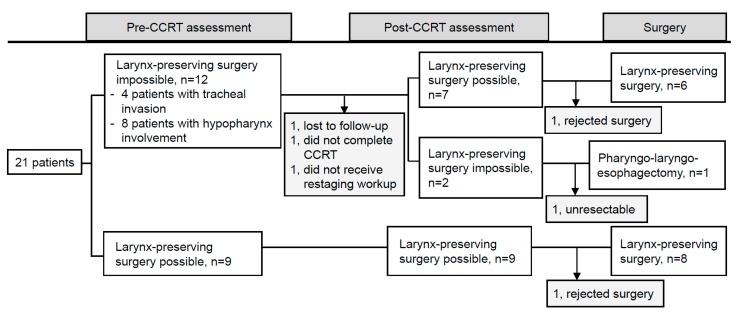
Flow chart to demonstrate the study population and the practicability assessment of larynx preservation.

**Figure 2:**
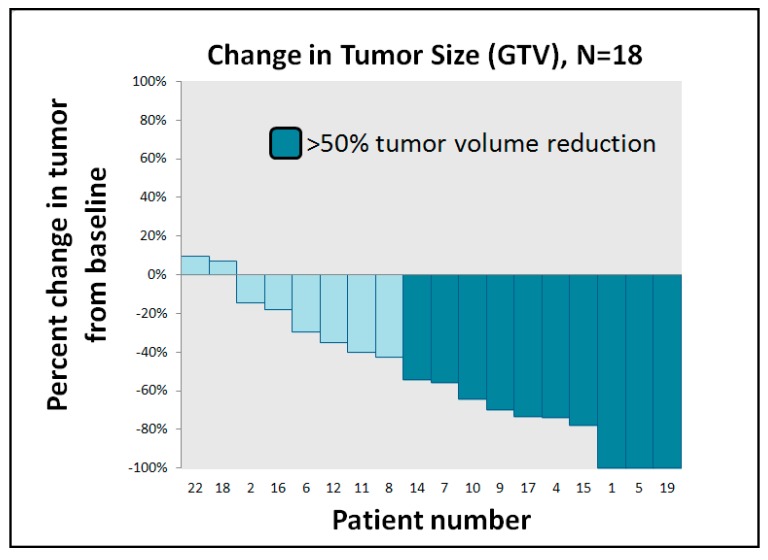
Waterfall plots for clinical tumor response. Gross tumor volumes (GTVs) were contoured to calculate tumor volume. Post-concurrent chemoradiotherapy (CCRT) GTVs were compared with pre-CCRT GTVs for treatment response evaluation.

One patient developed pneumonia and bacteremia with septic shock on the tenth day after neoadjuvant CCRT and died the same day. One patient was lost to follow-up at the completion of CCRT. One patient was considered unresectable case because of esophageal tumor persistent invasion to trachea, and this patient received salvage RT boost to gross tumor with a total dose to 60 Gy. Among the other 17 patients suitable for radical surgery, two patients refused surgery after neoadjuvant CCRT and sequential RT boost of 12–16 Gy was administered to the residual gross tumor. Overall, 15 patients received radical resection (Figure 1). Figure 2 illustrates GTV response assessed after neoadjuvant CCRT. Three patients achieved clinical radiographic complete response, and seven patients experienced radiographic tumor volume reduction to less than 50% of initial volume. Pathological T down-staging was noted in seven patients, and pathological complete response was achieved in three of 15 patients. Fourteen of these 15 patients (93.3%) preserved the larynx and eight patients (53.3%) achieved R0 resection. Among 5 R1 resection patients, all patients had presence of tumor cells within 1 mm of the circumferential resection margin but with adequate axial margins of at least 5 mm. We added adjuvant chemotherapy for four of the five patients classified as R1 resection. Two patients had positive macroscopic resection margins because the tumors contacted the trachea. 

### 3.4. Surgical Complications

Complications after surgery are listed in Table 3. One patient died of colon graft failure and anastomotic leakage 1 month after surgery. This patient had a clinical stage of T4b. After neoadjuvant CCRT, the objective tumor response was stable disease. During surgery, the residual tumor at the posterior membrane portion of the trachea was still present. Esophagectomy was done but with residual gross tumor. The other surgery-related complications included abdominal wound problems, chylothorax, and pneumonia, which were manageable.

### 3.5. Treatment Outcomes

**Figure 3:**
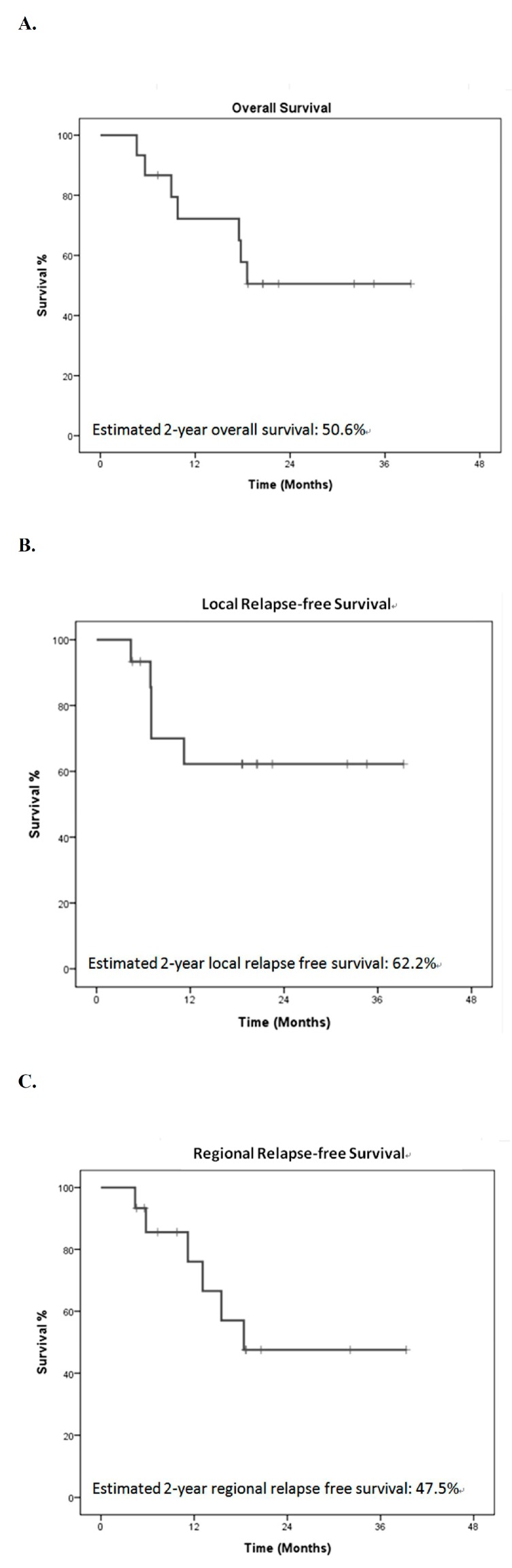
Survival. Kaplan–Meier plot of survival in patients who received neoadjuvant chemoradiotherapy followed by surgery for cervical esophageal cancer. (**A**) overall survival, (**B**) local relapse-free survival, (**C**) regional relapse-free survival.

At the time of analysis, the median follow-up time was 13.7 months (range, 1.0–46.9 months). For patients who underwent neoadjuvant CCRT followed by surgery for CEC, preliminary results indicate 2-year overall survival, local relapse-free survival, and regional relapse-free survival by the Kaplan–Meier method of 50.6%, 62.2%, and 47.5%, respectively (Figure 3). Among five R1 resection patients, three patients developed local recurrence, one patient experienced pulmonary metastasis without evidence of locoregional recurrence, and one patient was in disease-free status during the follow-up period.

## 4. Discussion

CECs are uncommon diseases, and the current standard for its treatment remains unknown [5,6]. CECs usually behave aggressively and are often locally advanced at initial presentation. Surgery is usually not performed because the tumors often infiltrate many complicated structures adjacent to the cervical esophagus. If radical surgery is performed, total pharyngo-laryngo-esophagectomy is usually inevitable, a procedure that includes removal of larynx, which has a significantly detrimental impact on the quality of life.

In previous decades, new surgical techniques to preserve the larynx have been developed. Various methods of reconstruction, including free jejunal graft, colon interposition, gastric tube pull-up, and pectoralis major myocutaneus flap have been demonstrated [24,25,26,27]. Nevertheless, the basic criterion for successful larynx preservation surgery is that the hypopharynx is not involved. Therefore, patients with a high esophageal tumor invading the hypopharynx are usually not considered suitable candidates for larynx-preserving surgery. Larynx preservation in patients with high CEC involving the hypopharynx might increase the duration of cannulation and raise the risk of persistent aspiration, resulting in fatal pneumonia [27].

To improve the quality of life in these patients, dCRT has been adopted as a primary treatment for CEC. The use of dCRT is based primarily on the results from clinical trials for non-CEC, such as Radiation Therapy Oncology Group 85-01 [28]. Although this study enrolled only patients with thoracic esophageal cancer, the results formed the current basis of dCRT for patients with esophageal cancer, including CEC. Several published studies investigated dCRT for patients with CEC, but most have been retrospective and heterogeneous in techniques (Table 4). In general, dCRT achieved 5-year overall survival rates ranging from 19% to 55% for patients with CEC, preserving their laryngopharyngeal function. However, most early series reported that dCRT resulted in unsatisfactory local control rates of 33% to 69% in advanced CEC even with RT dose escalation. Burmeister et al. reported a high local control rate of 88% in patients treated with dCRT, which may be due to the early stage of disease, as the majority of patients (71%) were stage I or IIA [29].

The benefits of adding surgery to chemoradiotherapy when compared to dCRT for nonmetastatic esophageal cancer are still unclear. A Cochrane review analyzed dCRT versus chemoradiotherapy plus surgery for esophageal cancer [30]. There is moderate-quality evidence that adding surgery to chemoradiotherapy probably increases the time to locoregional relapse (HR 0.55, 95% CI 0.39–0.76). However, this may be at the cost of increased treatment-related morality with no improvement in overall survival (HR 0.99, 95% CI 0.79–1.24). It is important to note that the participants of included studies were patients with cancer of thoracic esophagus, thus the aforementioned conclusion is uncertain in CEC. Valmasoni et al. analyzed the outcomes of CEC patients according to the treatment that included surgery alone, dCRT, or dCRT followed by surgery [31]. For patients with complete clinical response after CCRT, there was no survival benefit in the dCRT followed by surgery group compared with the dCRT group (*p* = 0.176). In contrast, surgery improved the survival significantly after dCRT (*p* = 0.023) in clinical non-complete responders with acceptable surgical morbidity and mortality. The limitation in current situation is that there is no consensus for the diagnosis of clinical complete response after CCRT. Given the high local failure rate with chemoradiotherapy and the difficulty to predict a pathological complete response even with repeat survey, trimodality treatment still has a role to play in CEC.

Given that surgery might be the solution for durable local-regional tumor control and that larynx preservation is the key issue concerning quality of life, the development of a new strategy to promote both is of critical importance. Neoadjuvant CCRT followed by radical surgery could be assessed as a larynx-preservation strategy for CEC on the basis of current progress in surgical and reconstructive techniques and RT delivery methods, including IMRT and helical tomotherapy. For surgical techniques, the development of robotic-assisted surgery and multidisciplinary surgical teams may also augment the resectability of CEC.

Our preliminary results showed that 14 of 15 (93.3%) CEC patients undergoing surgery had successful larynx preservation after neoadjuvant CCRT, even though near half had hypopharynx involvement initially. Achievement of negative resection margins has been reported to be a significant prognostic factor [22,23] and 8 patients (53.3%) had R0 resection in this study.

The definition of positive microscopic circumferential resection margin (CRM) in esophageal cancer differs between two major organizations of pathologists. The Royal College of Pathologists (RCP) considers the CRM to be positive if the tumor is located within 1 mm of the surgical margin [32], whereas the College of American Pathologists (CAP) defines a positive CRM as tumor found at the cut margin of resection [33]. We conservatively used RCP criteria to define positive CRM. However, the optimal definition of R1 CRM related to prognosis remains unclear. If we use CAP criteria, these 5 patients would be regarded as negative CRM and classified as R0 resection. Of the five patients with R1 resection, all had adequate proximal resection (longitudinal) margin but positive (axial) CRM. Further radical surgery, such as total pharyngo-laryngo-esophagectomy to resect whole larynx may not significantly change the status of CRM.

To successfully perform esophagectomy with sparing larynx, the minimal distance from distant end of pyriform sinus is 2 cm. We performed larynx-sparing esophagectomy with resection line at this minimally required line for tumor with shrinkage down to this line, no matter the initial tumor proximal end was cross this line or not. It is possible that if we determine the proximal resection line according to proximal tumor end detected before neoadjuvant CCRT, the local recurrence rate might be improved especially for initial proximal tumor end below 2 cm from distant end of pyriform sinus.

In this study, the acute toxicity for neoadjuvant CCRT followed by larynx preservation surgery was moderate but acceptable. One patient died during CCRT, which was not regarded as treatment-related mortality. Late adverse events were almost negligible, with the majority of patients experiencing esophageal stenosis. Most could be relieved by esophageal dilatation treatment. Because median follow-up time is still short (13.7 months; range, 1.0–46.9 months), other late toxicity profiles, such as hypothyroidism or cardiovascular disease, remain to be monitored.

The incidence of anastomotic leak reported in the literature varies between 5 and 40% following esophageal resection and anastomosis. A meta-analysis showed cervical anastomosis could be associated with a higher leak rate than intrathoracic anastomosis (odds ratio 3.43, 95% CI 1.09–10.78) [34]. The incidence of cervical anastomotic leak in the present study post-neoadjuvant CCRT is 13.3% and is considered acceptable. When proximal resection line is located at initial invaded region received planned full dose of RT, the risk of leakage becomes higher. Multiple factors which may be associated with anastomotic leak include number of preoperative comorbidities, levels of hemoglobin and albumin, location of tumor, type of conduit, anastomotic technique (hand sewn versus stapled), institutional and surgeon volume, and sepsis. Aggressive interventions to maintain adequate hemoglobin and albumin and prompt identification and treatment of infection perioperatively are encouraged to reduce the risk of anastomotic leak.

There are some important limitations of this study. This is a retrospective analysis of consecutive case series at a single institution, which indicates that this study is vulnerable to several types of bias and confounders. The small number of patients and the short follow-up period require caution when interpreting the statistical analysis. In addition, the weekly cisplatin regimen used in this study may compromise the response rate of neoadjuvant CCRT. The most common concurrent chemotherapy is typically composed of fluorouracil and cisplatin or carboplatin (AUC of 2 mg/mL per minute) and paclitaxel (50 mg/m2) in a neoadjuvant CCRT setting [21,28]. Because the cervical esophagus is near the hypopharynx, our study used weekly cisplatin based on experience with head and neck cancer as it is expected to be safer and less toxic. Patients receiving low-dose weekly cisplatin can be monitored for toxicity more often, allowing the schedule to be adjusted if needed. However, emerging evidence shows that this regimen might be suboptimal and should not be considered the standard of care [35,36].

## 5. Conclusions

In conclusion, the preliminary results of our study were comparable with those of historical studies using definitive CCRT or surgery for CEC. Neoadjuvant CCRT with subsequent larynx-sparing surgery for CEC can be considered a new treatment option in selected operable candidates with good performance status. Prospectively designed studies in patients with a longer follow-up duration for validation of this multimodality treatment in cervical esophageal SCC are warranted.

## Figures and Tables

**Table 1:** Patient characteristics.

Parameters	Number of Patients
Median age (years)	57 (range: 40*–*82)
Female/Male	0/21
Performance status (ECOG 0–1/2)	21/0
Clinical stage (I/II/III/IV) ^a^	0/6/15/0
T status (1/2/3/4)	1/3/12/5
N status (0/1/2/3)	5/9/6/1
M status (0/1)	21/0
Tumor extension	
CE	1
HP+CE	7
CE+TE	8
HP+CE+TE	5
Resectable/unresectable before CCRT ^b^	17/4
Concurrent chemotherapy (with/without)	21/0

^a^ Classification of the clinical stage was based on the seventh edition of the TNM classification for esophageal cancer. ^b^ All unresectable patients had tumor invading the trachea. Abbreviations: ECOG, Eastern Cooperative Oncology Group; CE, cervical esophagus; HP, hypopharyngeal extension; TE, thoracic esophageal extension.

**Table 2:** Acute toxicities in patients who underwent neoadjuvant concurrent chemoradiotherapy (CCRT) (*n* = 21).

	Grade 0 ^a^	Grade 1	Grade2	Grade 3	Grade 4
Pneumonitis	20	0	1	0	0
Cough	9	4	7	1	0
Esophagitis	9	7	5	0	0
Dermatitis	8	12	1	0	0
Anemia	1	12	7	1	0
Leukocytopenia	9	7	4	1	0
Neutropenia	16	2	2	1	0
Thrombocytopenia	16	3	2	0	0
Nausea	10	10	1	0	0
Vomiting	17	4	0	0	0

^a^ Grade per Common Terminology Criteria for Adverse Events version 4.0.

**Table 3:** Surgical complications in patients who underwent surgery for cervical esophageal cancer.

Complication	Numbers of Patient (*n* = 15)
Abdominal wound problem	3
Neck wound infection	1
Pharyngocutaneous fistula	1
Anastomotic leakage	2
Graft failure	1
Pneumonia	2
Chylothorax	1
Operative mortality	1

**Table 4:** Results of definitive CCRT and surgery for cervical esophageal cancer (CEC) in previous studies.

**Definitive CCRT for Cervical Esophageal Cancer**							
Author	Patients, No.	Con-CT, %	Dose of RT, Gy	Treatment-Related Mortality (%)	LC, %	Overall Survival (%)	
						2-Year	5-Year
Stuschke et al. 1999 [11]	17	Yes ^a^	60–66 Gy	0	33 (2 y) ^b^	24	NA
Burmeister et al. 2000 [29]	34	Yes, 100	50.4–65 Gy (mean 61.2)	5.9	88	NA	55
Yamada et al. 2006 [13]	27	Yes, 85.2	44–73.7 Gy(mean 66)	NA	52	NA	37.9
Wang et al.2006 [37]	22 (13) ^c^	Yes ^d^	24.5–64.8 Gy (median 50.4)	NA	47.7 (5 y) ^c^	NA	18.6 ^c^
Uno et al. 2007 [38]	21	Yes, 90.5	60–74 Gy (median 64)	4.8	NA	41	27
Huang et al. 2008 [39]	21	Yes	54 Gy/20 fx	NA	48 (2 y)	52	NA
	29	Yes	70 Gy/30 fx	NA	46 (2 y)	43	NA
Tong et al. 2011 [12]	21	Yes, 100	60–68 Gy	4.8	NA	46.9	NA
Gkika et al. 2013 [40]	55	Yes, 100	50–70 Gy (median 60)	0	55 (2 y) ^b^	35	25
Cao et al. 2014 [5]	161	Yes, 23.4	59.4–80 Gy	NA	69.9 (2 y)	51	NA
Grass et al. 2015 [4]	240	NA	NA	NA	NA	40	28
Cao et al. 2015 [15]	115	Yes, 30	59.4–80 Gy	1.7	68.3 (2 y)	47.6	NA
Zhang et al. 2015 [14]	102	Yes, 100	50–70 Gy	0	35.3 (3 y) ^e^	NA	NA
Cao et al. 2016 [16]	64	Yes, 34.4	60–80 Gy (median 62)	1.6	74.5 (2 y)	42.5	NA
Herrmann et al. 2017 [41]	55	Yes, 92.7 ^f^	28–72 Gy (median 56)	0	52 (3 y) ^b^	NA	NA
Zhao et al. 2017 [42]	86	Yes, 70	50–70 Gy (median 61.6)	0	57.9 (3 y) ^e^	NA	NA
Li et al. 2018 [17]	92	Yes, 100	60 Gy	4.3	NA	66.3	NA
**Primary Surgery for squamous cell carcinoma (SCC) of the Cervical Esophagus**							
Author	Patients, No.	RT/CRT, %		Treatment-Related Mortality (%)	LC, %	Overall Survival (%)	
		Neoadjuvant	Adjuvant			2-Year	5-Year
Triboulet et al. 2001 [9]	78 (131) ^g^	12.9 ^g^	73 ^g^	4.8 ^g^	78 ^g^	NA	14
Daiko et al. 2007 [43]	74	0	14.9	4	51.4 ^b^	NA	33
Kadota et al. 2009 [27]	32	0	NA	0	79.1 (low-tumor group)71.8 (high-tumor group) (5 y)	NA	NA
Ott et al. 2009 [18]	109	86	0	2.8	70 ^h^	61.8	47
Tong et al. 2011 [12]	62	0	61	7.1	NA	37.6	NA
Cao et al. 2014 [5]	63	0	57.1	1.5	68.6 (2 y)	50.7	NA
Grass et al. 2015 [4]	32	44	31	NA	NA	64	43

^a^ All patients received induction chemotherapy followed by concurrent chemoradiotherapy. ^b^ Loco-regional control. ^c^ With upper thoracic esophageal cancer (upper thoracic esophageal cancer cases). ^d^ 17% of patients received induction chemotherapy. ^e^ Loco-regional failure-free survival. ^f^ 58.2% of patients received induction chemotherapy. ^g^ With hypopharyngeal cancer (hypopharyngeal cancer cases). ^h^ In patients who had a complete (R0) resection. Abbreviations: Con-CT, concurrent chemotherapy; LC, local control; NA, not available.
